# Progressive slowdown/prevention of cellular senescence by CD9-targeted delivery of rapamycin using lactose-wrapped calcium carbonate nanoparticles

**DOI:** 10.1038/srep43299

**Published:** 2017-04-10

**Authors:** Raj Kumar Thapa, Hanh Thuy Nguyen, Jee-Heon Jeong, Jae Ryong Kim, Han-Gon Choi, Chul Soon Yong, Jong Oh Kim

**Affiliations:** 1 College of Pharmacy, Yeungnam University, 280 Daehak-Ro, Gyeongsan, Gyeongsanbuk-do, 712-749, Republic of Korea; 2 Department of Biochemistry and Molecular Biology, College of Medicine, Yeungnam University, Daegu, 705-717, Republic of Korea; 3 College of Pharmacy, Hanyang University, 55, Hanyangdaehak-ro, Sangnok-gu, Ansan 426-791, Republic of Korea

## Abstract

Cellular senescence, a state of irreversible growth arrest and altered cell function, causes aging-related diseases. Hence, treatment modalities that could target aging cells would provide a robust therapeutic avenue. Herein, for the first time, we utilized CD9 receptors (overexpressed in senescent cells) for nanoparticle targeting in addition to the inherent β-galactosidase activity. In our study, CD9 monoclonal antibody-conjugated lactose-wrapped calcium carbonate nanoparticles loaded with rapamycin (CD9-Lac/CaCO_3_/Rapa) were prepared for targeted rapamycin delivery to senescent cells. The nanoparticles exhibited an appropriate particle size (~130 nm) with high drug-loading capacity (~20%). *In vitro* drug release was enhanced in the presence of β-galactosidase suggesting potential cargo drug delivery to the senescent cells. Furthermore, CD9-Lac/CaCO_3_/Rapa exhibited high uptake and anti-senescence effects (reduced β-galactosidase and p53/p21/CD9/cyclin D1 expression, reduced population doubling time, enhanced cell proliferation and migration, and prevention of cell cycle arrest) in old human dermal fibroblasts. Importantly, CD9-Lac/CaCO_3_/Rapa significantly improved the proliferation capability of old cells as suggested by BrdU staining along with significant reductions in senescence-associated secretory phenotypes (IL-6 and IL-1β) (*P* < 0.05). Altogether, our findings suggest the potential applicability of CD9-Lac/CaCO_3_/Rapa in targeted treatment of senescence.

Cellular senescence refers to a state of irreversible growth arrest and altered function of normal somatic cells after a finite number of divisions^1^. Senescent cells are characterized by a flattened shape, senescence-associated β-galactosidase (SA-β-gal) activity, and hypersecretion of cytokines, chemokines, and proteases^2^,^3^. Senescence partly depends on mechanistic target of rapamycin (mTOR) signaling^2^ that mainly regulates tumor suppressor pathways p53/p21 and Rb/p16^4^, and leads to disease development/progression through tissue function impairment. In addition, progressive inability of the immune system to destroy senescent cells during aging results in the accumulation of “death-resistant” cells that accelerate aging and disease development by altering neighboring cell behavior, lowering the pool of mitotic-competent cells, degrading the cellular matrix, and stimulating cancer^5^. Diverse age-related diseases such as atherosclerosis, muscle atrophy, skin aging, and benign prostate hyperplasia result from cellular senescence progression^6^. Therefore, strategies for the prevention, treatment, or removal of senescent cells are of prime interest for clinical applications.

A recently reported proof-of-concept demonstrated the use of capped mesoporous silica nanoparticles for targeted cargo delivery inside senescent cells mediated by β-galactosidase activity^5^. However, it fails to justify cell-specific uptake of these nanosystems to senescent cells following intravenous or subcutaneous delivery. A mechanism driven approach for specific interaction and uptake of nanoparticles by senescent cells has thus become a challenging necessity. Hence, we proposed a proof-of-concept regarding delivery of rapamycin (Rapa) loaded calcium carbonate (CaCO_3_) nanoparticles with CD9 receptor mediated targeting, in addition to utilization of β-galactosidase activity, in senescent cells.

Rapamycin (Rapa), an mTOR inhibitor, was found to prevent replicative senescence in rat embryonic fibroblasts by affecting the p53/p21 pathway^7^. In addition, several studies have indicated the beneficial effects of Rapa for life span extension in aging models^8^,^9^. More importantly, CD9-a glycoprotein receptor of the tetraspanin family that regulates cellular activity, development, growth, and motility^10^ - is overexpressed in senescent cells and thus, can potentially be used in targeted drug delivery. Although contradictory reports on CD9 receptors in different cancer cells suggest either enhancement^11^,^12^ or inhibition of growth and motility functions^13^,^14^, implying cell type-specific activity, senescent cells are closely related to cancer development. Our study is the first report for the utilization of CD9 receptors in targeting drug-loaded nanoparticles to senescent cells and can be a stepping stone for further research in the field of targeted therapy to senescent cells.

Nanoparticles (NPs) are widely investigated for targeted and controlled drug release^15^. Calcium carbonate (CaCO_3_) NPs exhibit advantages (e.g., high availability, minimal toxicity, easy surface modification, and slow biodegradability) that warrant their use in controlled drug delivery^15^. In this study, therefore, we prepared CD9 monoclonal antibody (mAb)-conjugated CaCO_3_ NPs aimed at specific targeting of senescent cells. The NPs were wrapped with lactose-polyethylene glycol (Lac-PEG) conjugate (CD9-Lac/CaCO_3_/Rapa) for stabilization in the blood circulation, prevention of opsonization, and targeted cargo release mediated by β-galactosidase. β-galactosidase breaks down lactose, thereby unwrapping the outer layer of the NPs favoring drug release for treatment of senescence.

## Results and discussion

**Figure 1:**
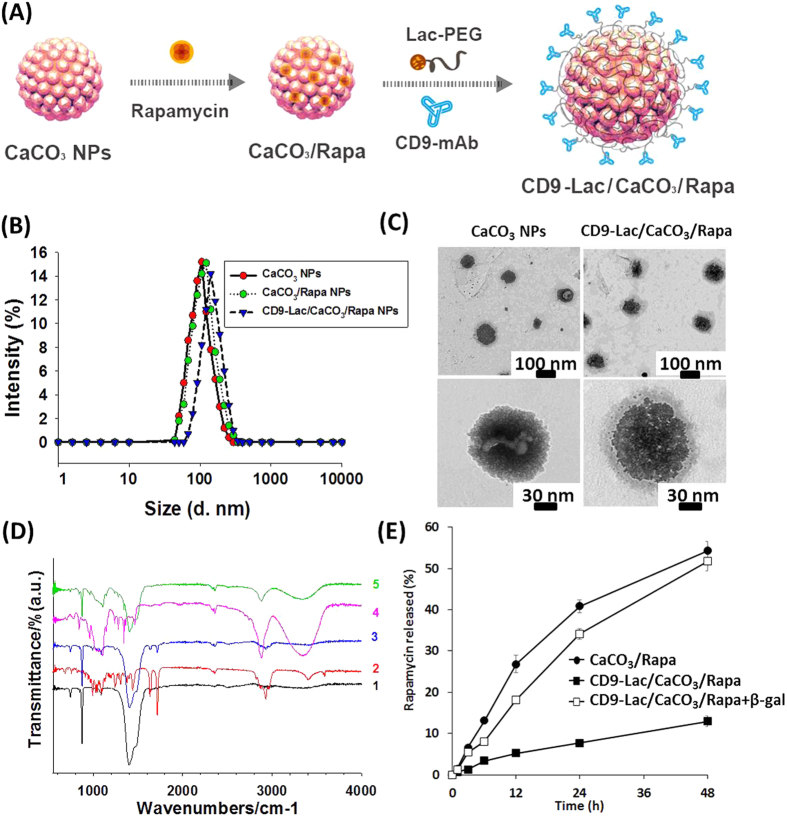
(**A**) Schematic representation for CD9 mAb-conjugated Lac/CaCO_3_/Rapa NPs. (**B**) Particle size distribution, and (**C**) TEM images of CD9-Lac/CaCO_3_/Rapa NPs. (**D**) FTIR spectra. 1: CaCO_3_ NPs, 2: Rapa, 3: CaCO_3_/Rapa, 4: Lac-PEG-COOH, 5: CD9-Lac/CaCO_3_/Rapa. (**E**) *In vitro* drug release profiles of Rapa from different formulations at pH 7.4 (β-gal: β-galactosidase).

As a first step in CD9-Lac/CaCO_3_/Rapa NP fabrication, CaCO_3_ NPs were successfully prepared (Fig. 1A). Dynamic light scattering (DLS) measurements revealed that the CaCO_3_ NPs had an average size of ~100 nm (Fig. 1B), which was confirmed by transmission electron microscopy (TEM) (Fig. 1C) and atomic force microscopy (AFM) (Fig. S1, Supplementary Information). The surface charge of the NPs became more positive with decreasing pH and *vice versa* (Fig. S2A, Supplementary Information), which can be attributed to the protonation of carbonate ions^16^. Subsequently, Rapa was incorporated into the CaCO_3_ NPs. The addition of 0.2 mg of Rapa per mg of CaCO_3_ NPs yielded the highest encapsulation efficiency (EE) and loading capacity (LC) (Fig. S2B, Supplementary Information). Therefore, this concentration was used in further experiments. Rapa loading can be ascribed to the porous structure of the NPs that prevented any alterations in the particle sizes of NPs after drug loading^17^.

The next step in NP optimization involved the wrapping of CaCO_3_/Rapa with Lac-PEG-COOH conjugate (Fig. S2C, Supplementary Information) and the conjugation of CD9 mAb through EDC/NHS chemistry. The CD9-Lac/CaCO_3_/Rapa NPs had a slightly increased size of ~130 nm as indicated by DLS, TEM, and AFM (Figs 1B,C, S1, Supplementary Information), indicating successful conjugate layer wrapping. The presence of the conjugate layer slightly reduced Rapa EE and LC (Fig. S2D, Supplementary Information). The NPs were further characterized by fourier transform infrared spectroscopy (FTIR) (Fig. 1D). Blank CaCO_3_ NPs presented major spectral bands at 1405 and 857 cm^−1^ corresponding to ʋ3-asymmetric stretching vibrations and the Ca–O stretching vibration of the CO_3_^2−^ group, respectively^18^. Pure Rapa revealed characteristic peaks at 1000–1800 cm^−1^ and 2800–3000 cm^−1^. The CaCO_3_/Rapa spectrum was similar to that of blank NPs, suggesting successful incorporation of Rapa within the NP pores. In the CD9-Lac/CaCO_3_/Rapa spectrum, the intensity of the CaCO_3_-specific bands was dramatically reduced while characteristic Lac-PEG-COOH bands were clearly observed, suggesting successful wrapping of the NPs with the conjugate. Conjugation of CD9 mAb to the NPs was confirmed by SDS-PAGE (Fig. S3, Supplementary Information). Non-reduced and reduced CD9 mAb produced characteristic bands in the gel. Following conjugation with the NPs, the non-reduced form of CD9 mAb did not shift while under reducing conditions, the characteristic bands were observed, suggesting successful conjugation. *In vitro* drug release of Rapa from the NP formulations was analyzed in PBS (pH 7.4 containing 1.0% Tween 80) using a dialysis method (Fig. 1E). Rapa release from CD9-Lac/CaCO_3_/Rapa and CaCO_3_/Rapa was compared with or without β-galactosidase. Drug release from CD9-Lac/CaCO_3_/Rapa was significantly retarded as compared to CaCO_3_/Rapa, which can be attributed to the hindering effect of the conjugate layer. Importantly, the presence of β-galactosidase significantly accelerated drug release from CD9-Lac/CaCO_3_/Rapa by breaking down lactose into smaller units^19^.

**Figure 2:**
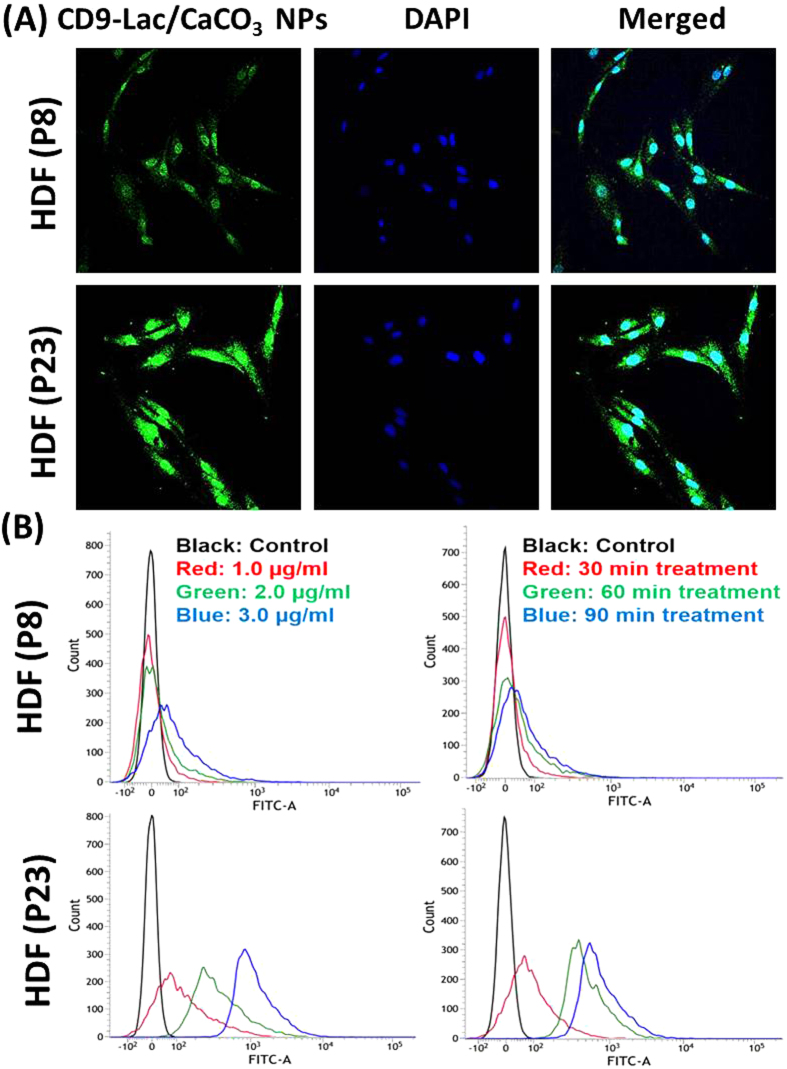
(**A**) Confocal images showing cellular uptake of CD9-Lac/CaCO_3_ NPs. Quantitation of cellular uptake of CD9-Lac/CaCO_3_ NPs in (**B**) a concentration-dependent and (**C**) a time-dependent manner. Coumarin 6 was used as a fluorescent probe (Scale bar: 30 μm).

**Figure 3:**
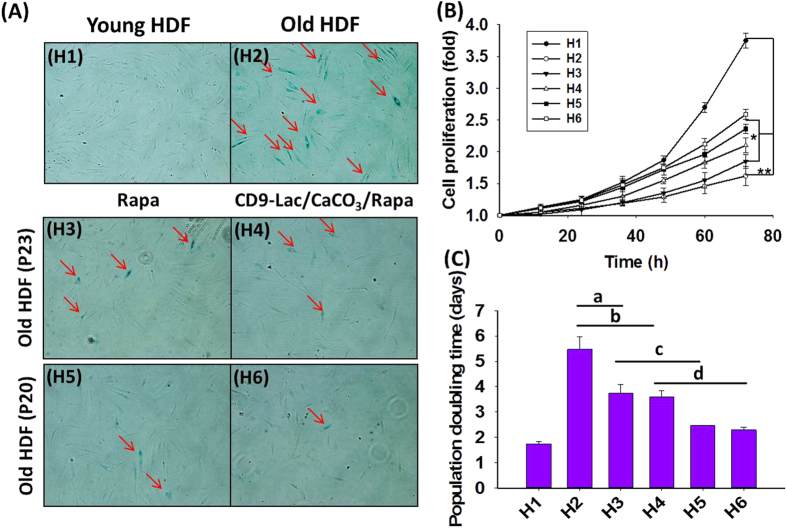
(**A**) Microscopic images of young and old β-galactosidase-stained HDFs. Arrows indicate β-galactosidase positive cells. (**B**) Cell proliferation (^*^P < 0.05, ^**^P < 0.01) and (**C**) population doubling times for young and old HDFs (a, b, c, d: *P* < 0.05). (H1) Young HDFs (P8), (H2) old HDFs (P23), (H3) old HDFs of P23 treated with Rapa, (H4) old HDFs of P23 treated with CD9-Lac/CaCO_3_/Rapa NPs, (H5) old HDFs of P20 treated with Rapa, and (H6) old HDFs of P20 treated with CD9-Lac/CaCO_3_/Rapa NPs,. HDFs in (H3), (H4), (H5), and (H6) were treated with Rapa or CD9-Lac/CaCO_3_/Rapa NPs for 72 h, followed by 3 passages without additional treatment.

We used HDFs of different passages as models of normal and senescent cells. HDFs of passage ≤10 (P8) were used as young cells while HDFs of passage ≥20 (P23) were regarded old. P20 was regarded as early senescence and P23 as late senescence based on cell proliferation and population doubling times. First, we determined the CD9 expression levels in young and old HDFs. Immunofluorescence and western blot analyses revealed significantly higher expression of CD9 receptors in old than in young cells (Fig. S4, Supplementary Information), corroborating our hypothesis that CD9 receptors can be used for targeted cargo delivery to senescent cells. Next, we compared CD9-Lac/CaCO_3_ NP uptake by young and old HDFs using coumarin-6 loaded CD9-Lac/CaCO_3_ NPs. As expected, old HDFs presented higher cellular NP uptake than young HDFs as demonstrated by confocal microscopy, most likely because of enhanced CD9 expression by old HDFs (Fig. 2A). Further quantitative assessments by FACS revealed higher uptake in both concentration- and time-dependent manner only in old HDFs (Fig. 2B). Senescent cells characteristically present high β-galactosidase expression and reduced cell proliferation^2^. As indicated by β-galactosidase staining, young HDFs did not express β-galactosidase while old cells highly expressed the enzyme (Fig. 3A). Furthermore, young cells displayed a very high rate of cell proliferation, which progressively reduced with increased passaging of cells (P20–P23) (Fig. S5A, Supplementary Information). Conversely, the population doubling time was minimal for young HDFs and increased from P20 to P23 (Fig. S5B, Supplementary Information). Together, these results indicated cell cycle arrest, typical of the G1 phase in which DNA replication is inhibited.

Next, we investigated whether CD9-Lac/CaCO_3_/Rapa were able to slow down/prevent senescence by comparing β-galactosidase expression and cell proliferation in HDFs (P20 and P23) treated or not with free Rapa or CD9-Lac/CaCO_3_/Rapa (6 experimental groups in total). HDFs were treated with free Rapa or CD9-Lac/CaCO_3_/Rapa for 72 h, followed by 3 passages before analysis because the proliferative effect of Rapa was found to be high after 3 passages (Fig. S6, Supplementary Information). β-galactosidase expression in HDFs is presented in Fig. 3A. The number of HDFs (P23) positive for β-galactosidase staining clearly decreased following treatment with Rapa or CD9-Lac/CaCO_3_/Rapa, which can be attributed to mTOR inhibitory activities of Rapa. The effect was even more significant for HDFs (P20). CD9-Lac/CaCO_3_/Rapa was more effective than free Rapa because of controlled Rapa release from the NPs inside the cells, while free Rapa could be easily effluxed by senescent cells, possibly through enhanced Pgp1 receptor expression^20^. The effect of CD9-Lac/CaCO_3_/Rapa treatment in terms of improvement of cell proliferation was larger in HDFs (P20) than in HDFs (P23) (Fig. 3B). These results suggest that pre-treatment of cells progressing towards senescence (early senescence) with targeted delivery of Rapa could efficiently inhibit the aging process and revive the cells as compared to treatment of cells in the senescent state (late senescence). Similarly, the population doubling time was signficantly reduced in free Rapa- or CD9-Lac/CaCO_3_/Rapa-treated HDFs (P23) when compared to untreated old HDFs (Fig. 3C). Additionally, CD9-Lac/CaCO_3_/Rapa-treated HDFs (P20) showed significantly reduced population doubling times when compared to free Rapa-treated HDFs.

**Figure 4:**
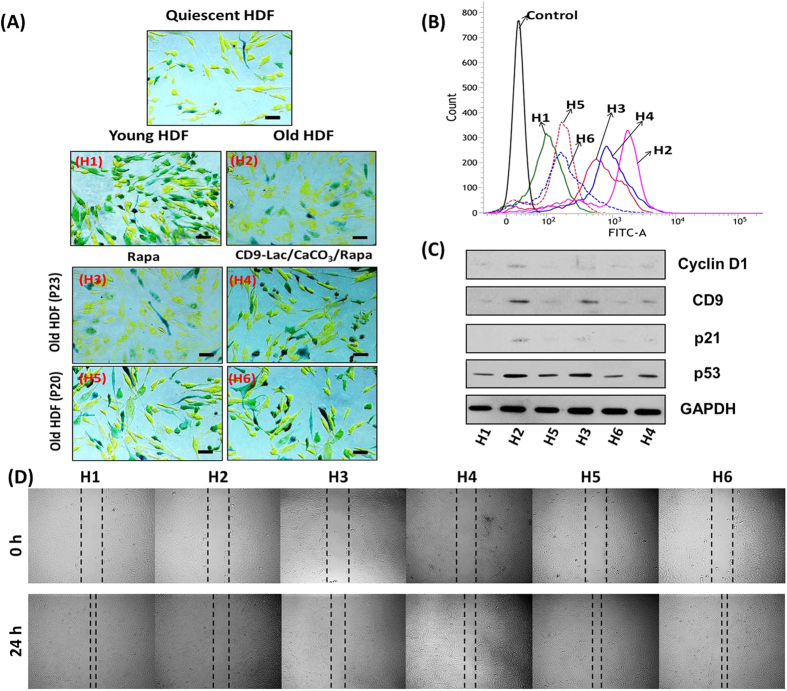
(**A**) Effects of free Rapa and CD9-Lac/CaCO_3_/Rapa NPs treatments on cell cycle of young and old HDFs (Scale bar = 100 μm). Quiescent HDFs, prepared by serum starvation for 48 h, were used as a control. Determination of: (**B**) ROS levels in young and old HDFs; (**C**) expression of the indicated proteins; and (**D**) cell migration of young and old HDFs. (H1) Young HDFs (P8), (H2) old HDFs (P23), (H3) old HDFs of P23 treated with Rapa, (H4) old HDFs of P23 treated with CD9-Lac/CaCO_3_/Rapa NPs, (H5) old HDFs of P20 treated with Rapa, and (H6) old HDFs of P20 treated with CD9-Lac/CaCO_3_/Rapa NPs,. HDFs in (H3), (H4), (H5), and (H6) were treated with Rapa or CD9-Lac/CaCO_3_/Rapa NPs for 72 h, followed by 3 passages without additional treatment.

Senescent cells are predominantly characterized by activation of the growth-promoting TOR pathway and blockage of the cell cycle resulting in cellular hypertrophy^21^. We determined the effect of Rapa treatment on the cell cycle in old HDFs using the Cell Clock^TM^ assay, which uses a redox dye that changes color based on the cell cycle phase (G1, S, G2, and M) (Fig. 4A). Young HDFs comprised cells from all four phases whereas old HDFs predominantly comprised G1-phase cells, suggesting G1 phase cell cycle arrest. A comparative analysis suggested similar patterns of G1 phase cell cycle arrest between quiescent and old HDFs. Treatment of HDFs (P23) with CD9-Lac/CaCO_3_/Rapa moderately prevented this G1 cell cycle blockage through controlled Rapa release from NPs (Fig. S7, Supplementary Information). Free Rapa had very limited effect because of easy efflux from the old HDFs, likely caused by enhanced Pgp1 expression^20^. Importantly, treatment of HDFs (P20) with CD9-Lac/CaCO_3_/Rapa significantly inhibited cell cycle arrest with best activity.

Several studies have reported an essential role for ROS in the development of cellular senescence through the regulation of p53, p21, or p16 pathways^22^,^23^,^24^. In agreement with these studies, the ROS level in old HDFs was greatly enhanced as compared to that in young HDFs (Fig. 4B). Free Rapa and CD9-Lac/CaCO_3_/Rapa significantly reduced the cellular ROS levels in HDFs (P20), while the effect was limited in HDFs (P23). Old HDFs presented enhanced expression of p53, p21, CD9, and cyclin D1 as indicated by western blotting (Fig. 4C). Enhanced p53 and p21 are associated with development of cellular senescence^22^. Additionally, elevated levels of p21 in senescent cells are responsible for enhanced cyclin D1 expression, supporting the fact that p21 promotes nuclear cyclin D1 complex accumulation by inhibiting cyclin D1 nuclear export, resulting in G1 cell cycle arrest^25^. Following treatment of HDFs (P23) with CD9-Lac/CaCO_3_/Rapa, the expression of all these proteins was reduced and the effect was even more pronounced in HDFs (P20).

A common manifestation of aged skin is the delay in wound healing caused by combinational defects in epithelial regeneration, angiogenesis, and cell migration and proliferation^26^. Cell migration assay results of the young, old, and treated HDFs are presented in Fig. 4D. Young HDFs possessed a high cell migration ability, which was significantly reduced in old HDFs. Treatment of old HDFs (P20) with CD9-Lac/CaCO_3_/Rapa restored their cell migration ability, an effect mediated by Rapa. Treatment of cells in early senescence phase showed better anti-senescence effects than treatment of cells in the late senescence phase. Nevertheless, treatment of senescent HDFs (P23) with CD9-Lac/CaCO_3_/Rapa improved their condition.

**Figure 5:**
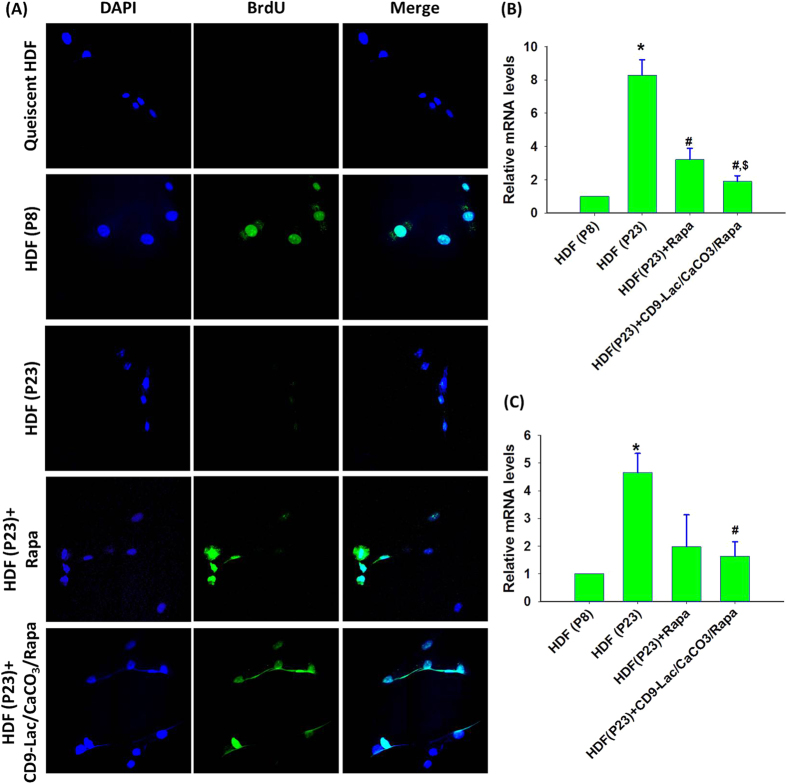
(**A**) Cell proliferation assay for HDFs stained with BrdU (green); DAPI was applied as a nuclear counter-staining dye (blue). Quiescent cells were used as a control for growth-arrested cells. Relative mRNA levels of (**B**) IL-6 and (**C**) IL-1β for HDFs [*P < 0.05 as compared to HDFs at P8; ^#^*P* < 0.05 as compared to HDFs at P23; ^$^*P* < 0.05 as compared to HDFs at P23 + Rapa].

A BrdU staining assay in HDFs (Fig. 5A) was performed using nuclear DAPI counterstaining to indicate the cell division stage^27^. Quiescent cells clearly showed cessation of division as determined by a lack of BrdU staining. HDFs at P8 contained actively dividing cells that were absent in HDFs at P23. Rapa treatment facilitated cell division as evidenced by increased BrdU staining in the cells. Furthermore, CD9-Lac/CaCO_3_/Rapa treatment enhanced BrdU staining in HDFs at P23, which was attributed to the targeted Rapa delivery within the cells for effective senescence treatment.

IL-6, a pleiotropic proinflammatory cytokine, is the most prominent cytokine of the secretory phenotype associated with DNA damage-induced senescence of fibroblasts^28^,^29^. Senescent cells can directly affect neighboring cells through IL-6 expression mediated by the IL-6R (gp80) and gp130 signaling complex expressed on the cell surface^30^. In the present study, IL-6 mRNA levels for HDFs significantly (*P* < 0.05) increased in old HDFs at P23 as compared to that in young HDFs at P8 (Fig. 5B). Treatment of old HDFs with Rapa significantly reduced the mRNA levels of IL-6 (*P* < 0.05). Furthermore, a significant (*P* < 0.05) reduction in IL-6 mRNA levels in CD9-Lac/CaCO_3_/Rapa-treated HDFs as compared to that with Rapa treatment was observed. Similarly, IL-1β is another interleukin signaling molecule found to be overexpressed in senescent fibroblasts^31^. It affects neighboring cells through cell-surface receptors by triggering the nuclear factor kappa B and activating protein 1 pathways^32^. In the present study, the mRNA levels of IL-1β significantly (*P* < 0.05) increased in HDFs at P23 compared to that in HDFs at P8 (Fig. 5C). Rapa treatment of old HDFs reduced IL-1β mRNA levels but there was no significant difference as compared to their levels in HDFs without treatment. However, treatment of old HDFs with CD9-Lac/CaCO_3_/Rapa significantly (*P* < 0.05) decreased the mRNA levels of IL-1β, suggesting the effectiveness of targeted Rapa release by the nanoparticle formulation for the treatment of senescence.

**Figure 6:**
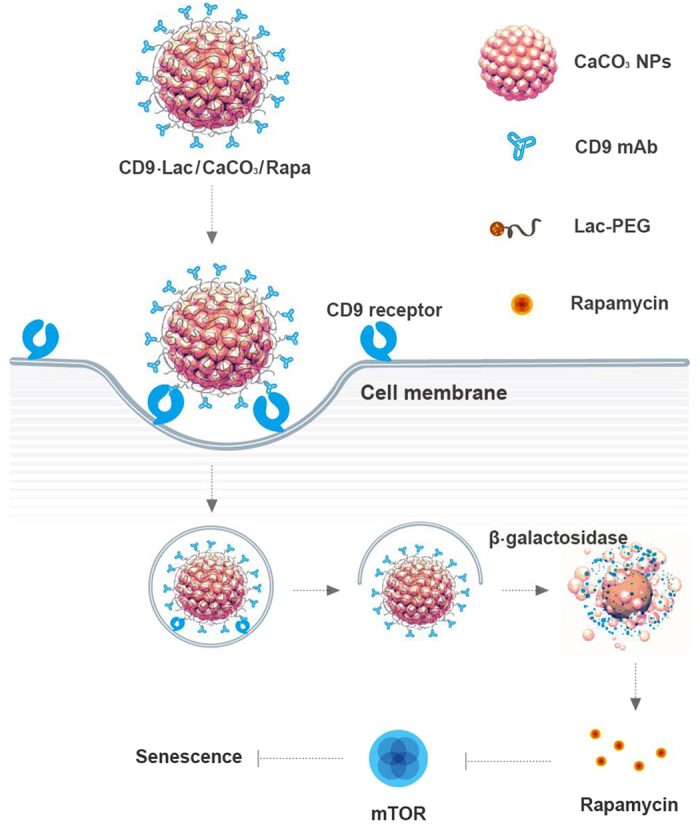
Schematic representation for CD9 receptor-targeted delivery of rapamycin to aging cells utilizing inherent β-galactosidase activity for lactose unwrapping and subsequent rapamycin release.

The results obtained for young and old HDFs treated with CD9-Lac/CaCO_3_/Rapa were further confirmed in an alternative senescent cell model. Doxorubicin-induced senescent cells were tested for uptake of these nanoparticles and subsequent amelioration of senescence. As presented in Fig. S8 (Supplementary Information), enhanced anti-senescent effects of CD9-Lac/CaCO_3_/Rapa as compared to those of Rapa were demonstrated by a reduction in β-galactosidase levels, enhanced uptake of nanoparticles in senescent cells, improved cell proliferation and population doubling times, and regulation of the cell cycle in senescent cells. Furthermore, BrdU incorporation and mRNA levels of IL-6 and IL-1β were evaluated for young, senescent, and treated cells (Fig. S9, Supplementary Information). As expected, Rapa significantly improved proliferation of and decreased the mRNA expression of IL-6 and IL-1β in senescent cells. Furthermore, significant improvements in BrdU incorporation and reduction in IL-6 and IL-1β levels were observed in senescent cells treated with CD9-Lac/CaCO_3_/Rapa. These results support the efficacy of targeted therapy of senescent cells using our novel nanoparticulate system. A schematic representation of the cellular uptake and fate of CD9-Lac/CaCO_3_/Rapa aimed at delivery of Rapa for progressive slowdown/prevention of senescence is presented in Fig. 6.

In summary, CD9 mAb-conjugated Lac/CaCO_3_/Rapa NPs were successfully fabricated for highly specific targeted drug delivery to senescent cells mediated by CD9 receptors. *In vitro* β-galactosidase-mediated Rapa release from the NPs suggested senescence-targeted drug delivery. The anti-senescence effects of CD9-Lac/CaCO_3_/Rapa in HDFs, including reduced β-galactosidase and p53/p21/CD9/cyclin D1 expression, enhanced cell proliferation and cell migration ability, reduced population doubling times, and prevention of cell cycle arrest, indicate the potential of the nanoformulation for progressive slowdown/prevention of cellular senescence. Furthermore, the enhanced proliferation ability and reduced IL-6 and IL-1β levels in CD9-Lac/CaCO_3_/Rapa-treated old HDFs indicate the potential anti-senescence effects of the targeted nano-formulation. Till date, very few studies utilizing NPs for therapy targeting senescence have been reported. In addition, there are no reports on receptor-targeted delivery of senolytics-loaded NPs to senescent cells. The current study provided proof-of-concept of CD9 receptor- and β-galactosidase activity-mediated targeted cargo delivery of NPs to senescent cells. Although preliminary, our results provide avenues for the effective delivery of senolytics, alone or in combination, for the elimination of senescent cells in potential rejuvenation therapies. We believe that our findings can serve as a stepping stone encouraging researchers for further in-depth studies utilizing our described NP model to develop treatments for existing senescence-related diseases.

## Methods

### Preparation of CD9-Lac/CaCO_3_/Rapa NPs

A double decomposition reaction was carried out to prepare the CaCO_3_ NPs by mixing 0.1 M of CaCl_2_·2H_2_O and NaHCO_3_, each prepared in water and ethylene glycol (1:5, v/v), followed by stirring for 3 h. Subsequently, the synthesized NPs were collected by sequential washing with ethanol, methanol, and acetone, followed by drying at 60 °C for 1 h. The CaCO_3_ nanoparticles were resuspended in distilled water supplement with an ethanolic solution of Rapa. The mixture was stirred for 3 h (to allow the loading of Rapa to porous NPs), followed by dialysis against distilled water containing 1.0% Tween 80 for further 24 h to obtain CaCO_3_/Rapa.

Separately, Lac-PEG-COOH conjugate was prepared using a 1:1 molar ratio of LAC and NH_2_-PEG-COOH. An ethanolic mixture of NH_2_-PEG-COOH was added to an aqueous suspension of lactose, stirred at room temperature for 24 h, dialyzed against distilled water, and freeze-dried to obtain LAC-PEG-COOH. FTIR analysis of Lac-PEG-COOH conjugate suggested that Lac presented characteristic peaks at 550–1400 cm^−1^ and 2800–3600 cm^−1^ (Fig. S2C). Similarly, NH_2_-PEG-COOH possessed characteristic peaks at 800–1800 cm^−1^ and 2800–3600 cm^−1^. The physical mixture mainly showed peaks characteristic of Lac whereas Lac-PEG-COOH conjugate revealed peaks characteristic of both Lac and NH_2_-PEG-COOH, suggesting successful synthesis of the conjugate.

Next, Lac-PEG-COOH was added to CaCO_3_/Rapa aqueous dispersion (at 2:1 w/w ratio) which was stirred for 3 h and then freeze-dried to obtain COOH-PEG-LAC/CaCO_3_/Rapa. Finally, a CD9-Lac/CaCO_3_/Rapa conjugate was produced by adding EDC and NHS to an aqueous COOH-PEG-Lac/CaCO_3_/Rapa dispersion followed by the addition of anti-CD9 antibody and mixing for 3 h.

### Characterization

#### Physicochemical characterization of CaCO_3_/Rapa and CD9-LAC/CaCO_3_/Rapa

Particle size, polydispersity index (PDI), and zeta potential of CaCO_3_, CaCO_3_/Rapa, and CD9-Lac/CaCO_3_/Rapa were analyzed using a Zetasizer Nano ZS (Malvern Instruments, Malvern, UK) equipped with Nano DTS software (version 6.34). Morphological analyses of CaCO_3_/Rapa and CD9-Lac/CaCO_3_/Rapa were carried out using TEM and AFM. TEM (H7600, Hitachi, Tokyo, Japan) analysis of carbon-coated copper grid loaded with CaCO_3_/Rapa or CD9-LAC/CaCO_3_/Rapa dispersion was conducted using 2% phosphotungstic acid solution as negative stain. Similarly, CaCO_3_/Rapa or CD9-LAC/CaCO_3_/Rapa deposited on ultraflat mica square plates (Ted Pella, Inc., USA) were used for AFM analysis with a Nanoscope^®^ IIIa Scanning Probe Microscope (Digital Instruments, USA). Furthermore, FTIR analyses of dried Lac-PEG-COOH conjugate and CD9-Lac/CaCO_3_/Rapa were carried out using a Thermo Scientific Nicolet Nexus 670 FTIR spectrophotometer.

#### Determination of drug loading

Drug-entrapment efficiency (EE, %) and drug-loading capacity (LC, %) were calculated by separating unbound drug from the drug-loaded NPs using an Amicon centrifugal filter device (molecular weight cut-off, 10,000 Da, Millipore). The prepared formulations were centrifuged at 5,000 rpm for 10 min to obtain unbound drug in the filtrate, which was analyzed using HPLC. HPLC system (Hitachi, Japan) was comprised of an L-2130 pump, L-2200 autosampler, L-2420 UV-Vis detector, and L-2350 column oven and was equiped with Ezchrom elite software (318a, Japan). An Inertsil C_18_ column (150 mm × 4.6 mm, 5 μm particle size, Cosmosil, Nacalai Tesque Inc., USA) was used for isocratic elution with a mobile phase comprising of 10 mM ammonium acetate:acetonitrile (30:70, v/v) at a flow rate of 1.0 mL/min and column temperature of 50 °C. A 20 μL sample was injected for each analysis and UV absorbance was measured at a wavelength of 278 nm. The following equations were used for the calculation of EE and LC:


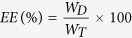


where, *W*_D_ = weight of formulation-bound Rapa, *W*_T_ = weight of total Rapa added to the formulation.


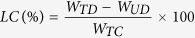


where, *W*_TD_, *W*_UD_, and *W*_TC_ are the weights of total Rapa, unbound Rapa, and total CaCO_3_, respectively.

#### In vitro drug release study

Release of Rapa from CaCO_3_/Rapa and CD9-LAC/CaCO_3_/Rapa in absence or presence of β-galactosidase was evaluated in phosphate-buffered saline (PBS, pH 7.4 containing 1.0% v/v Tween 80 to maintain sink condition) using a dialysis system comprised of Spectra/Por 3500 Da-MWCO membrane tubing. Release was studied at 100 rpm speed with a temperature maintained at 37 °C. Samples were withdrawn at specified time points and replaced with fresh media. The cumulative amount of Rapa release was calculated using HPLC as described above.

#### Immunocytochemistry

Cells were grown on coverslips in media and were fixed with 4% paraformaldehyde for 10 min. Then, the cells were washed thrice with PBS, permeabilized with 0.1% Triton X-100 for 15–20 min, and again washed thrice. Cells were then treated with 1% BSA in PBS (IH solution) for 45 min followed by incubation with anti-CD9 rabbit monoclonal antibody (1 μg/mL) in IH solution at 4 °C overnight. Then, the samples were thoroughly washed thrice with PBS and exposed to Alexa Fluor 488-labeled goat anti-rabbit IgG antibody (Thermo Scientific, USA). Fluorescence was measured using a laser scanning confocal microscope (Leica TCS SP8 STED 3X, Leica Microsystems, Germany).

#### Senescence induction

Unless noted otherwise, senescence was induced to HDFs by sequential passaging upto 20–23 passages leading to replicative senescence induction. Alternatively, cells were induced to senescence by incubation with doxorubicin (250 nM) for 3 days.

#### Cellular uptake study

HDFs (2 × 10^4^ cells/well) were seeded on cover slips placed in 12-well plates and incubated for 24 h. Coumarin-6-loaded CD9-LAC/CaCO_3_/Rapa NPs were added to each well and the cells were further incubated for 30 min. Then, the cells were washed with PBS and fixed with 4% paraformaldehyde in the dark. Finally, the cells were washed with PBS and the coverslips were carefully mounted on a glass slide, sealed with glycerin, and observed under the confocal laser-scanning microscope (Nanoscope Systems, Daejeon, Republic of Korea).

For quantitative measurement, HDFs (1 × 10^5^ cells/well) were seeded in 12-well plates and incubated for 24 h. The cells were treated with coumarin-6-loaded CD9-LAC/CaCO_3_/Rapa at different concentrations in a humidified incubator maintained at 37 °C with 5% CO_2_. At the indicated time points, the cells were washed with PBS, harvested, and dispersed in 0.5 mL of PBS solution for flow cytometric measurements using a FACSCalibur flow cytometer (BD Biosciences, USA).

#### Senescence-associated β-galactosidase staining

Senescence-associated β-galactosidase (SA-β-gal) activity in HDFs was evaluated as previously reported^33^. Images were taken with a DMIL LED microscope (Leica, Wetzlar, Germany).

#### Cell proliferation analysis

HDFs were seeded at 1 × 10^4^ cells/well in 6-well culture plates and treated with or without Rapa or CD9-Lac/CaCO_3_/Rapa for specified times. The cells were harvested and counted with a hemocytometer. Population doublings were calculated as previously described^34^.

#### Cell cycle analysis

Cell cycle analysis of HDFs was performed using the Cell Clock^TM^ Assay kit (Biocolor Ltd., UK), which can be used for live-cell detection and analysis of the four major phases (G1, S, G2, M) of the mammalian cell cycle during *in vitro* culture. Live HDFs in 12-well plates were treated with redox dye (Cell-Clock Dye Reagent) for 1 h at 37 °C and images were taken with the DMIL LED microscope. The percentage of cells in each phase was obtained from digitized photomicrographs using ImageJ software.

#### Determination of intracellular ROS levels

Intracellular ROS levels in HDFs were measured as described previously^35^. Intracellular H_2_-DCF fluorescence intensities of at least 10,000 cells were analyzed using the FACSCalibur flow cytometer.

#### SDS-PAGE

SDS-PAGE was carried out on 12% Tris-glycine gels. Stock protein samples were mixed with loading buffer and were electrophoresed with Tris-glycine running buffer for 2 h at 90 mV (Electrophoresis Power Supply; Thermo Scientific, Madison, WI, USA). The gel was stained with Coomassie blue for determination of the amount of NP-conjugated CD9 antibody.

#### Western blot analysis

Western blot analysis was performed using our previously reported method^36^. Antibodies against p53, p21, CD9, and cyclin D1 (Cell Signaling Technology, MA, USA) were used. Antigen-antibody complexes were detected with Western Blotting Luminol Reagent solution (Santa Cruz Biotechnology, CT, USA) on a LAS-3000 image system (Fujifilm, CT, USA).

#### In vitro cell migration assay

*In vitro* cell migration was assayed using a previously described method^37^. Multiple areas of the scratch wounds were photographed and the wound areas were compared.

#### 5-Bromo-2′-deoxyuridine (BrdU) assay

HDFs grown on round glass coverslips in 12 well plates were treated with BrdU for staining of the proliferating cells as described previously^6^. Images were obtained using a confocal laser scanning microscope (Nanoscope Systems, Daejeon, Republic of Korea).

#### Reverse transcription and real-time PCR

Total RNA was isolated from HDFs using TRI-solution (Bio Science Technology, South Korea) according to the manufacturer’s suggestion. Reverse transcription was carried out using 1 μg total RNA in a final reaction volume of 20 μL with MMLV reverse transcriptasae (Promega Corporation, Madison, WI, USA), 2.5 mM oligo-dT primers and 1 mM dNTPs. IL-6, IL-1β and GAPDH were amplified from cDNA with gene-specific primers (IL-6, forward: GCATGGGCCACCTCAGATTGT, reverse: TGCCCAGTGGACAGGTTTCT; IL-1β, forward: CACGGCCACATTTGGTTCTA, reverse: AGGGAAGCGGTTGCTCATC; GAPDH, forward: CGACCACTTTGTCAAGCTCA, reverse: AGGGGTCTACATGGCAACTG) and levels of each mRNAs were measured by real-time PCR using a SYBR Green PCR master mix with a 7500 Real-Time PCR system (Applied Biosystems, Foster City, CA, USA).

#### Statistical analysis

All the experimental results are expressed as the mean ± standard deviation (SD). Student’s t-test for pairs of groups and a one-way analysis of variance (ANOVA) for multiple groups were used to determine statistical differences. A p < 0.05 was considered statistically significant.

## Additional Information

**How to cite this article**: Thapa, R. K. *et al*. Progressive slowdown/ prevention of cellular senescence by CD9-targeted delivery of rapamycin using lactose-wrapped calcium carbonate nanoparticles. *Sci. Rep.* **7**, 43299; doi: 10.1038/srep43299 (2017).

**Publisher's note:** Springer Nature remains neutral with regard to jurisdictional claims in published maps and institutional affiliations.

## Supplementary Material

Supplementary Information
