# Supplementary material for: Progressive slowdown/prevention of cellular senescence by CD9-targeted delivery of rapamycin using lactose-wrapped calcium carbonate nanoparticles
Source: Sci Rep. 2017 Apr 10;7:43299. doi: 10.1038/srep43299 (PMC5385881; doi:10.1038/srep43299)
Supplement: Supplementary Information [file srep43299-s1.pdf]

Supplementary Information

Progressive slowdown/prevention of cellular senescence by CD9-targeted delivery of rapamycin using lactose-wrapped calcium carbonate nanoparticles

Raj Kumar Thapa[1], Hanh Thuy Nguyen[1], Jee-Heon Jeong[1], Jae Ryong Kim[2], Han-Gon Choi[3], Chul Soon Yong[1**], and Jong Oh Kim[1*]

[1]College of Pharmacy, Yeungnam University, 280 Daehak-Ro, Gyeongsan, Gyeongsanbuk-do, 712-749, Republic of Korea

[2]Department of Biochemistry and Molecular Biology, College of Medicine, Yeungnam University, Daegu, 705-717, Republic of Korea

[3]College of Pharmacy, Hanyang University, 55, Hanyangdaehak-ro, Sangnok-gu, Ansan 426-791, Republic of Korea

*Corresponding author: Prof. Jong Oh Kim, Ph.D.

Tel: +82-53-810-2813

Fax: +82-53-810-4654

E-mail: jongohkim@yu.ac.kr

** Co-corresponding author: Prof. Chul Soon Yong, Ph.D.

Tel: +82-53-810-2812

Fax: +82-53-810-4654

E-mail: csyong@yu.ac.kr

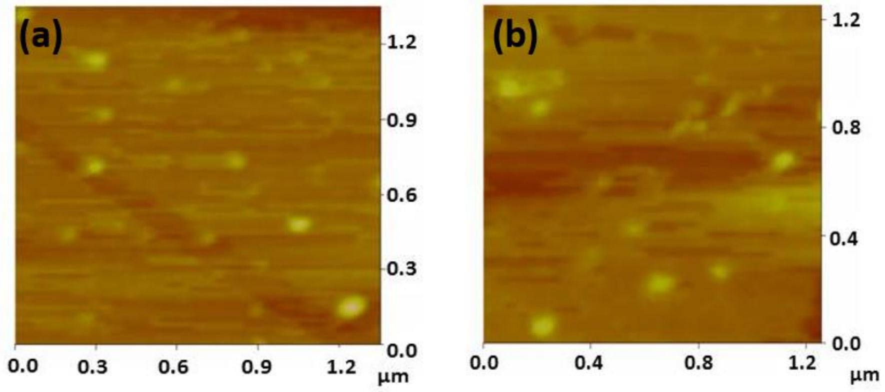

Figure S1: AFM images for (a) $CaCO_3$ NPs and (b) CD9-Lac/$CaCO_3$/Rapa NPs.

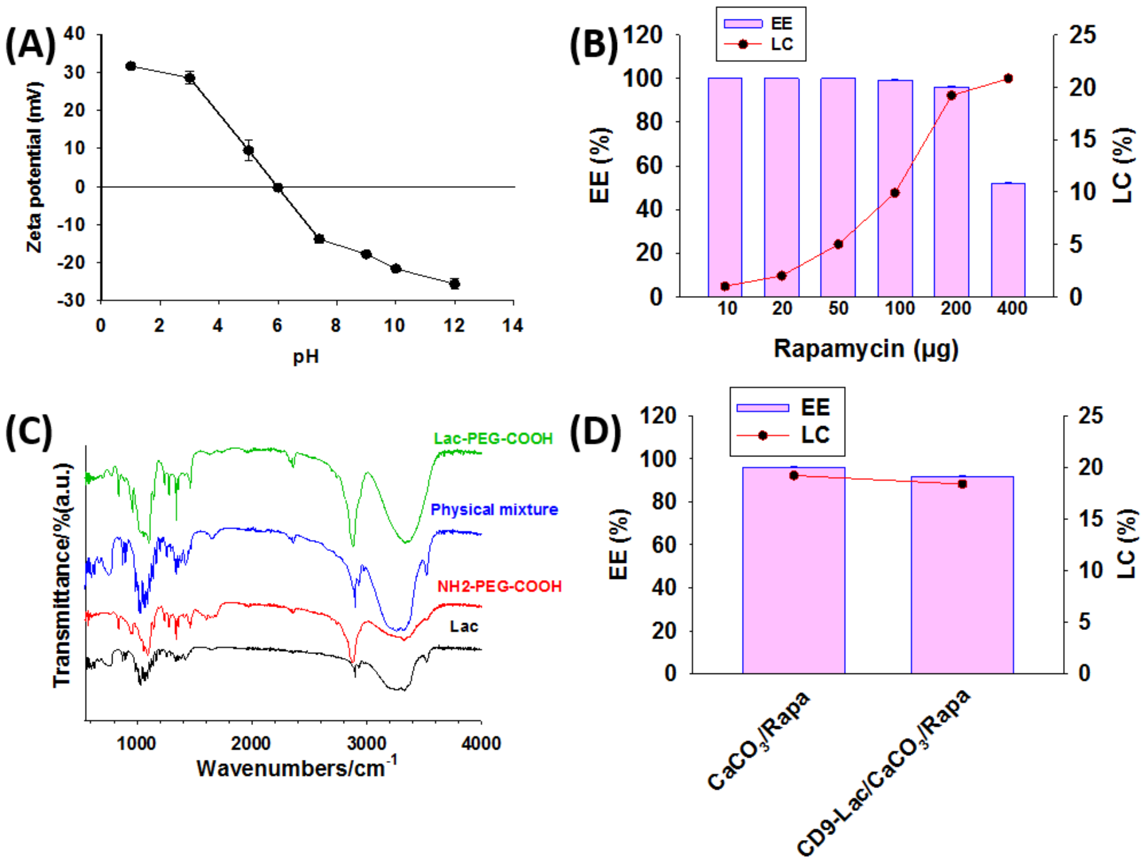

Figure S2: (A) Zeta potential of $CaCO_3$ NPs at different pH conditions. (B) EE and LC of Rapa in $CaCO_3$ NPs following addition of it's different amounts. (C) FTIR analysis of Lac-PEG-COOH conjugate. (D) EE and LC of Rapa.

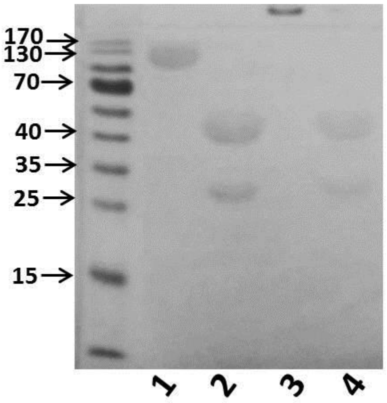

Figure S3: SDS-PAGE analysis for the determination of CD9 conjugation to PEG-Lac/$CaCO_3$/Rapa (1: non-reduced CD9 mAb; 2: reduced CD9 mAb; 3: non-reduced CD9 mAb conjugated as CD9-Lac/$CaCO_3$/Rapa; 4: reduced CD9 mAb conjugated as CD9-Lac/$CaCO_3$/Rapa).

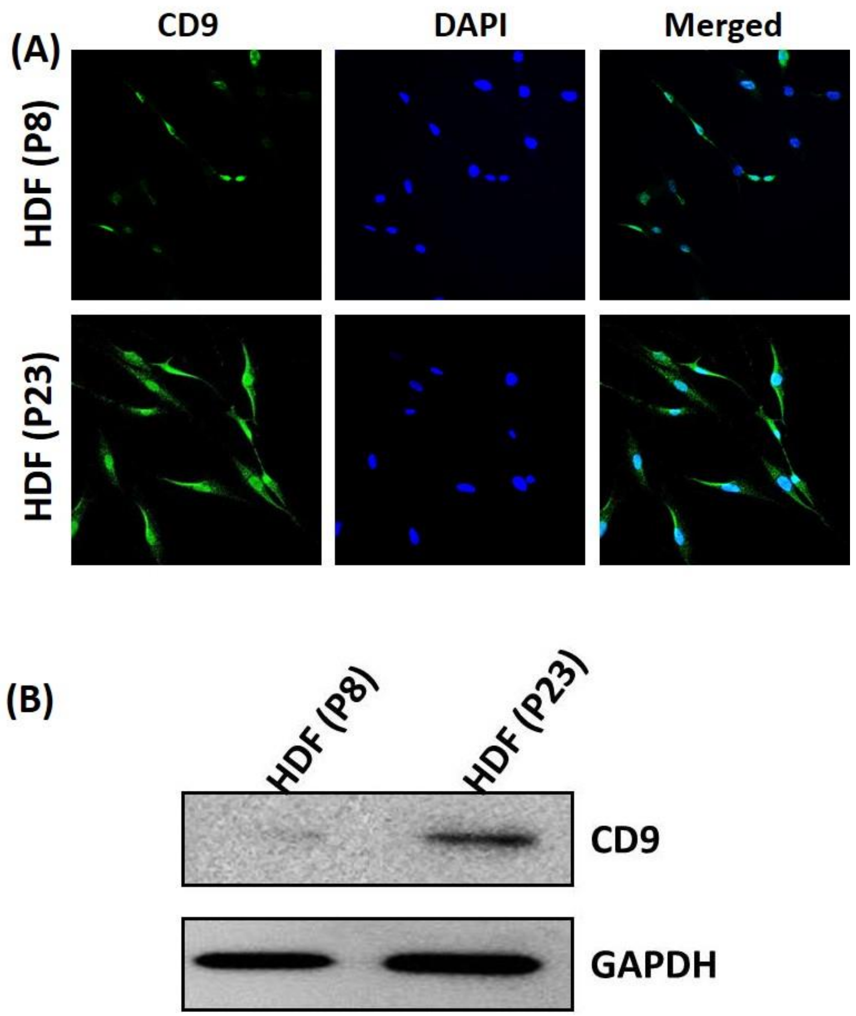

Figure S4: (A) Immunofluorescence analysis and (B) western blot analysis for the determination of CD9 receptor expression in young and old HDFs (Scale bar: 30 µm).

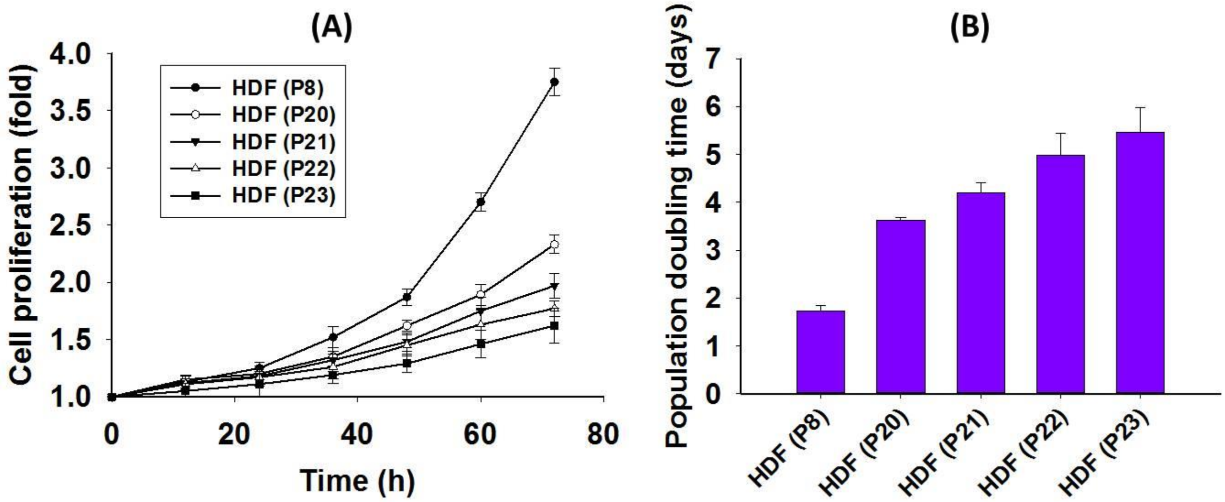

Figure S5: (A) Cell proliferation and (B) population doubling times in HDFs of different passages.

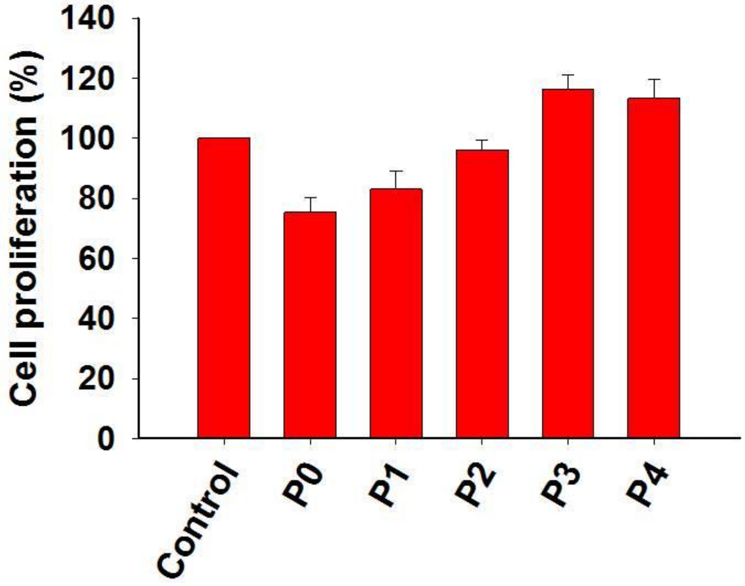

Figure S6: Cell proliferation of HDFs (P8) of different passages following treatment with Rapa (20 nM).

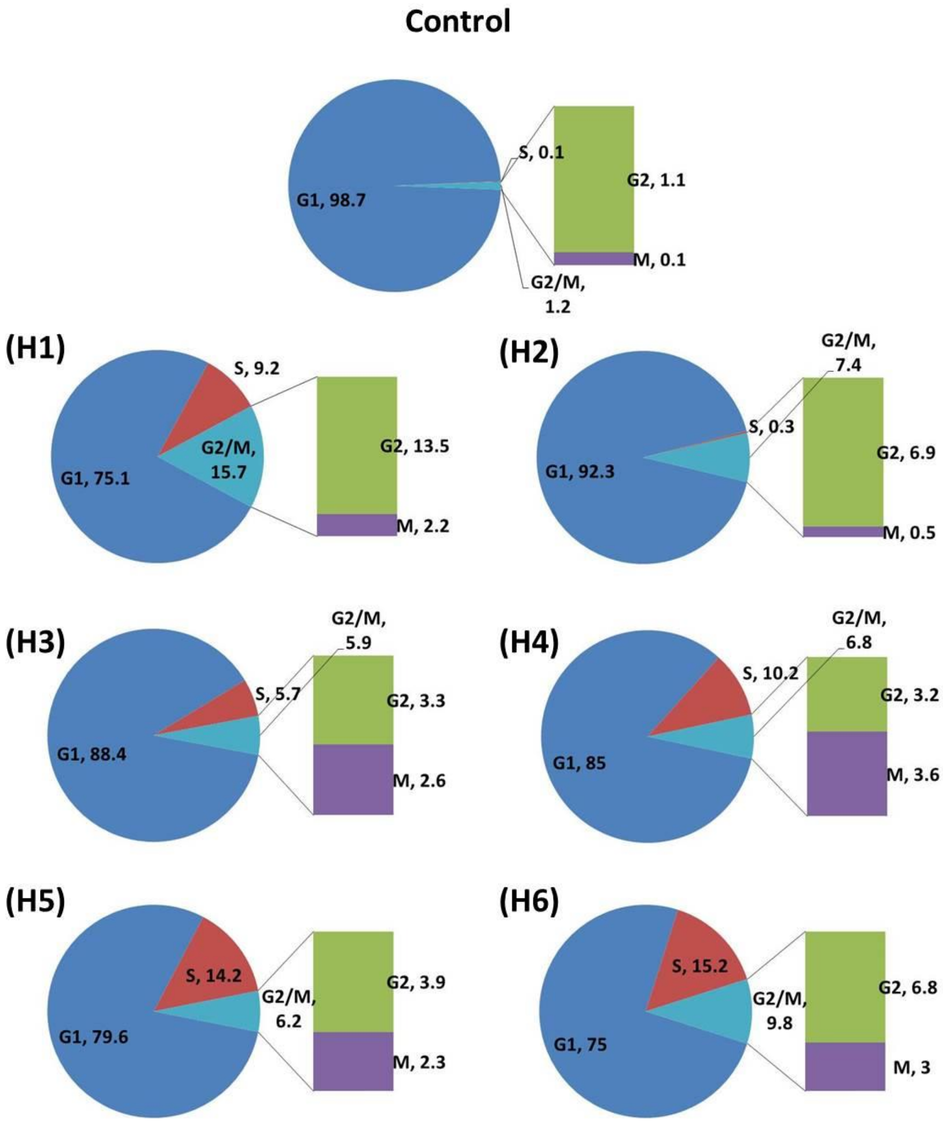

Figure S7: Effects of free Rapa and CD9-Lac/$CaCO_3$/Rapa NPs treatments on cell cycle of young and old HDFs Quiescent HDFs, prepared by serum starvation for 48 h, were used as a control. (H1) Young HDFs (P8), (H2) old HDFs (P23), (H3) old HDFs of P23 treated with Rapa, (H4) old HDFs of P23 treated with CD9-Lac/$CaCO_3$/Rapa NPs, (H5) old HDFs of P20 treated with Rapa, and (H6) old HDFs of P20 treated with CD9-Lac/$CaCO_3$/Rapa NPs,. HDFs in (H3), (H4), (H5), and (H6) were treated with Rapa or CD9-Lac/$CaCO_3$/Rapa NPs for 72 h, followed by 3 passages without additional treatment.

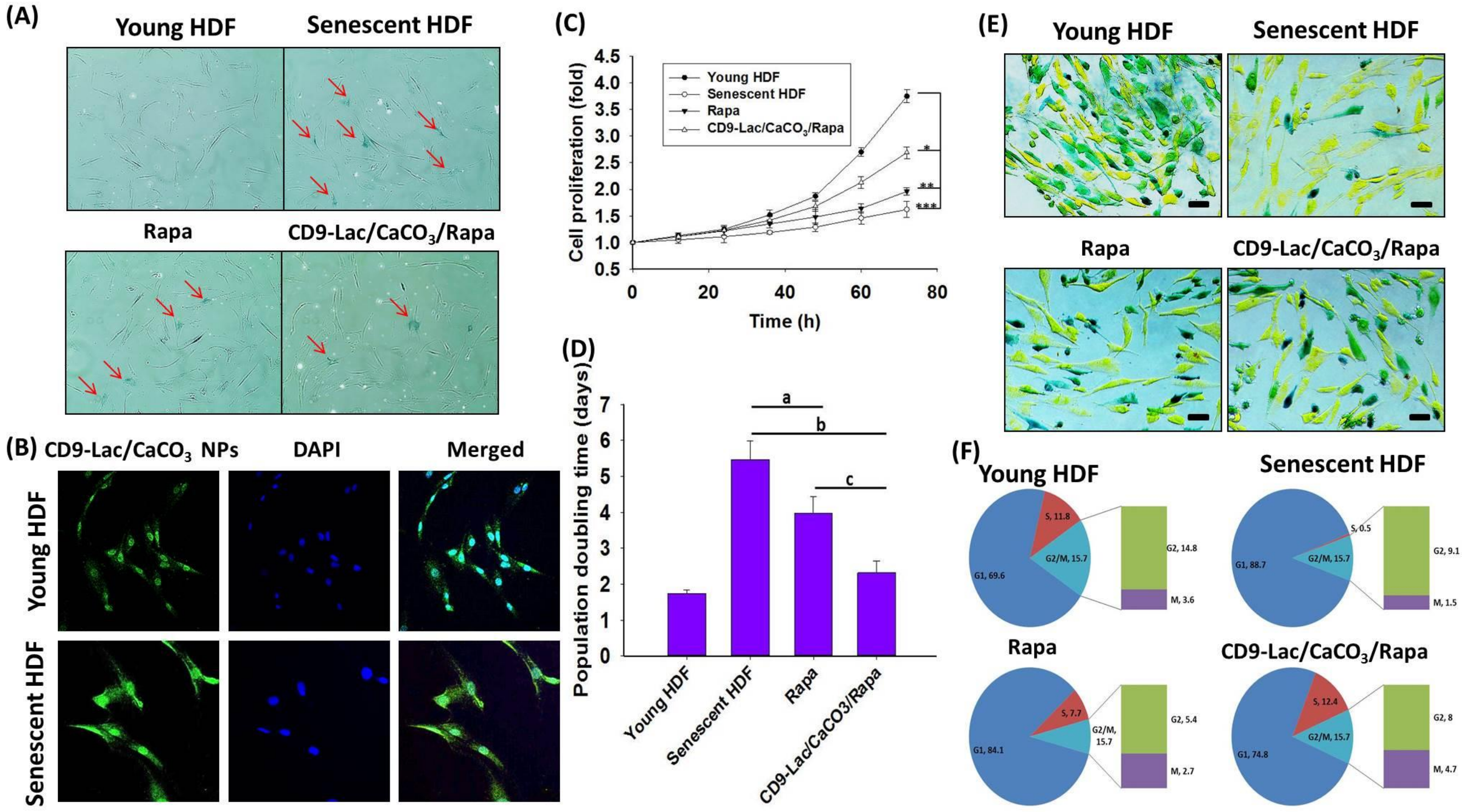

Figure S8: Determination of anti-senescence effects in an alternative senescence cell model. Doxorubicin (250 nM) treatment for 3 days was used to induce cellular senescence in HDFs. (A) Microscopic images of young and senescent β-galactosidase-stained HDFs. Arrows indicate β-galactosidase positive cells. (B) Confocal images showing cellular uptake of CD9-Lac/$CaCO_3$ NPs in young and

senescent HDFs (coumarin 6 was used as a fluorescent probe). (C) Cell proliferation ($^{*}P < 0.05$, $^{**}P < 0.01$, $^{***}P < 0.001$) and (D) population doubling times for young and senescent HDFs (a, b, c: $P < 0.05$). (E, F) Effects of free Rapa and CD9-Lac/$CaCO_3$/Rapa NPs treatments on cell cycle of senescent HDFs (Scale bar = 100 μm).

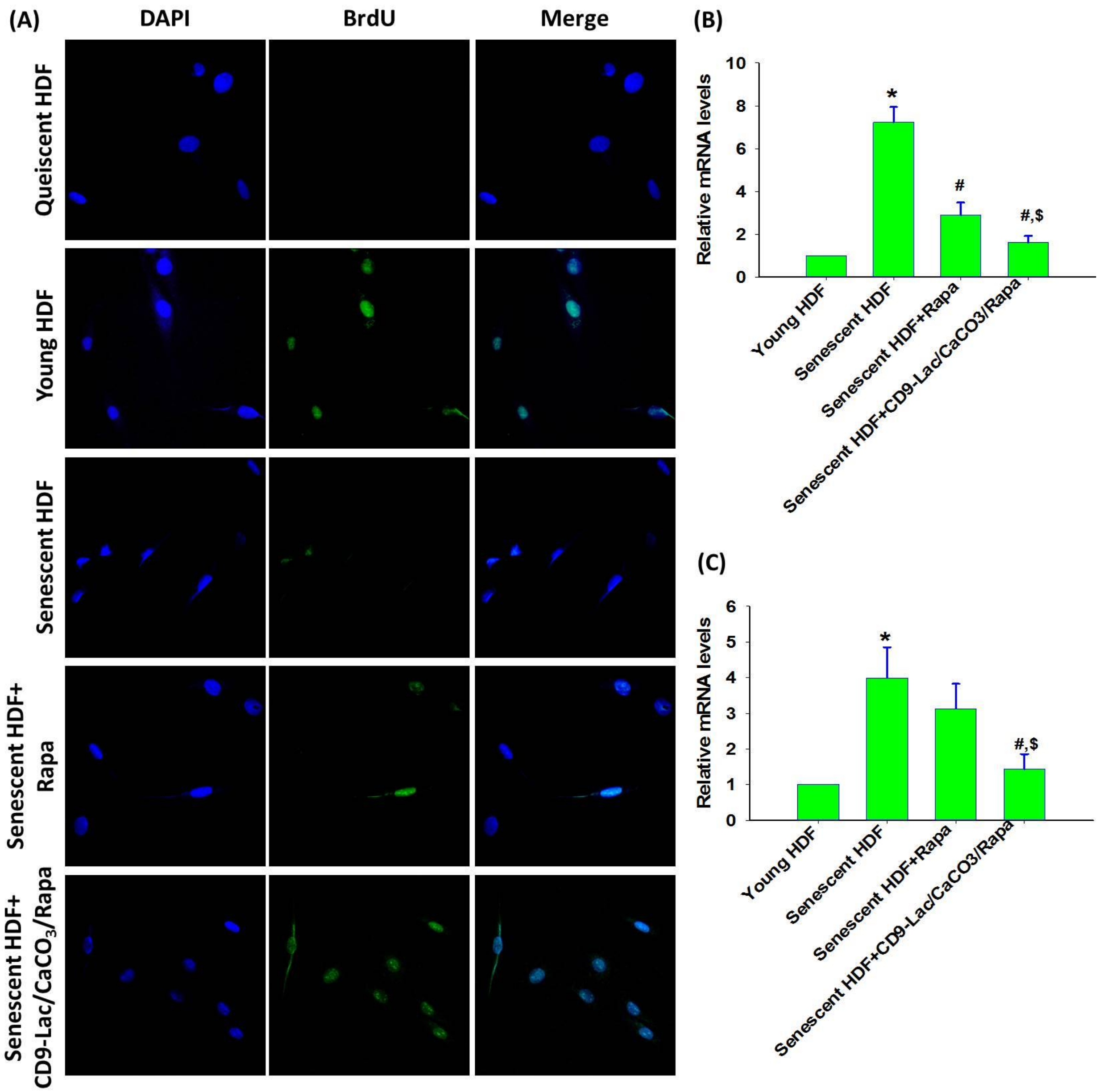

Figure S9: (A) Cell proliferation assay for HDFs stained for BrdU (green); DAPI was applied as nucleus counter staining dye (blue). Quiescent cells were used as control for growth arrested cells. Doxorubicin (250 nM) treatment for 3 days was used to induce cellular senescence in HDFs. Relative mRNA levels of (B) IL-6 and (C) IL-1β for HDFs [*: $P < 0.05$ as compared to young HDF; #: $P < 0.05$ as compared to senescent HDF; $: $P < 0.05$ as compared to senescent HDF+Rapa].
